# DR Congo and Nigeria: New neglected tropical disease threats and solutions for the bottom 40%

**DOI:** 10.1371/journal.pntd.0007145

**Published:** 2019-08-08

**Authors:** Peter Hotez

**Affiliations:** 1 Texas Children’s Hospital Center for Vaccine Development, Departments of Pediatrics and Molecular Virology and Microbiology, National School of Tropical Medicine, Baylor College of Medicine, Houston, Texas, United States of America; 2 Center for Medical Ethics and Health Policy, Baylor College of Medicine, Houston, Texas, United States of America; 3 Department of Biology, Baylor University, Waco, Texas, United States of America; 4 James A Baker III Institute for Public Policy, Rice University, Houston, Texas, United States of America; 5 Scowcroft Institute of International Affairs, Bush School of Government and Public Service, Texas A&M University, Texas, United States of America; University of Washington, UNITED STATES

New information indicates that two sub-Saharan Africa countries—the Democratic Republic of Congo (DR Congo) and Nigeria—will be home to almost one-half of the world’s population living in extreme poverty by 2050. The poverty-related neglected tropical diseases (NTDs) already disproportionately affect DR Congo and Nigeria, but by 2050, extreme poverty combined with political instability, food insecurity, urbanization, population shifts, and climate change could make these countries the epicenter of the world’s NTDs.

**Fig 1:**
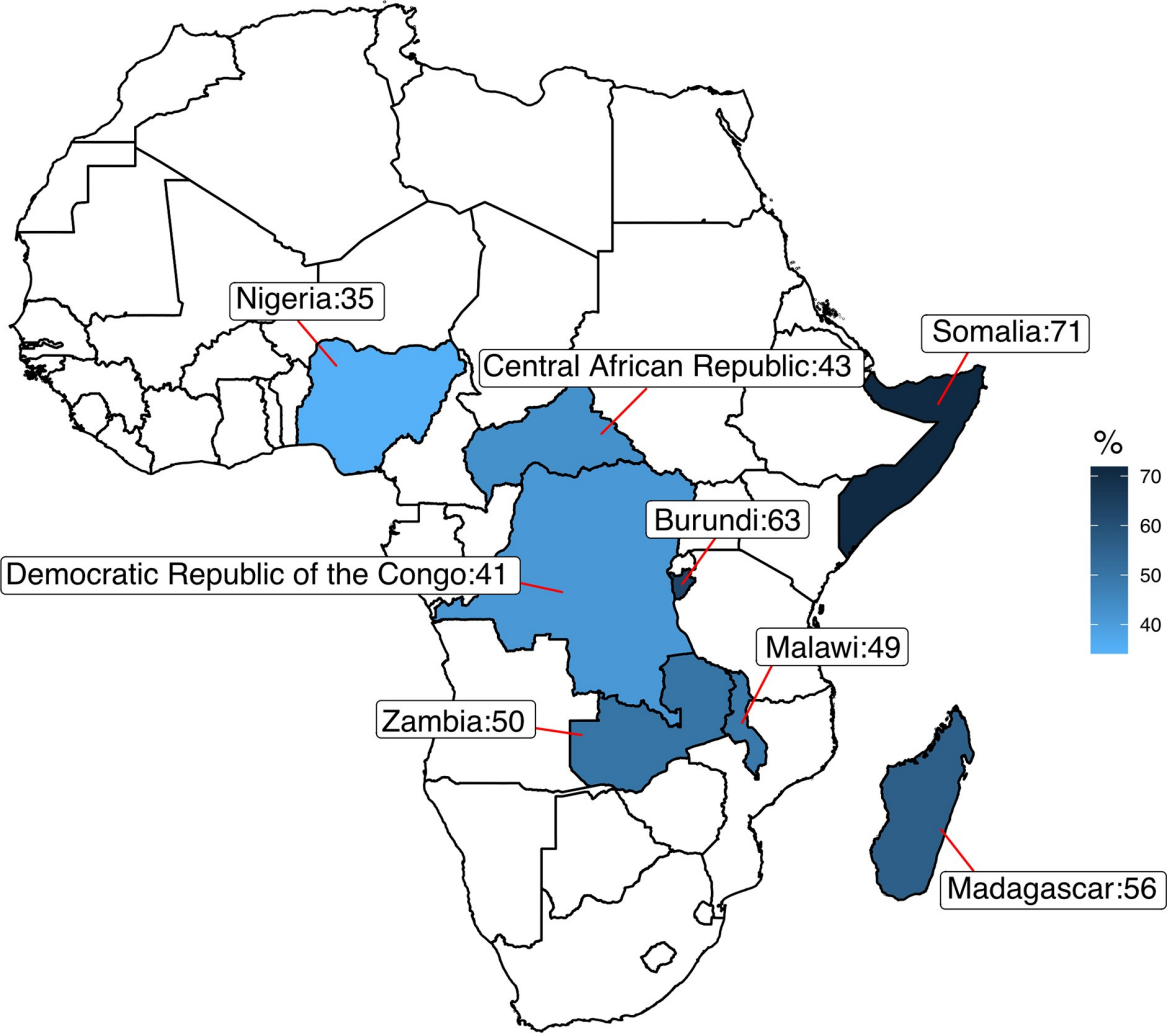
Projection of the percentage of the population living in extreme poverty in Africa by country (highest eight countries) in the year 2050. (Original figure made with data from [1].).

In the fall of 2018, the Bill & Melinda Gates Foundation and the Institute of Health Metrics and Evaluation at the University of Washington released an important “goalkeepers” report, focusing on great strides in poverty reduction—especially in China and elsewhere in Asia—but also highlighting the nations left behind and trapped in extreme poverty [1, 2]. One of the more striking findings was the prediction that by the year 2050, more than 40% of the world’s poorest people will be living in DR Congo and Nigeria [1]. The report finds that of the projected population of 429 million people in Nigeria, 152 million people (35%) will live in extreme poverty by 2050, as will 70 million of DR Congo’s projected population of 171 million (41%) (Fig 1) [1]. Additional “left behind” countries with high percentages of their population in extreme poverty could also include Burundi, Central African Republic, Madagascar, Malawi, Somalia, and Zambia [1].

Poverty is considered the overriding social determinant of the NTDs, with NTDs reinforcing poverty through their disabling effects [3]. Not surprisingly, our previous analysis conducted with the Global Burden of Disease Study (GBD) 2013 revealed that both DR Congo and Nigeria have the highest total numbers of people affected by selected NTDs, especially those endemic to Africa [4]. For example, the GBD 2013 found that globally, Nigeria has the largest number of cases of schistosomiasis, the second largest number of cases of onchocerciasis, and the third largest number of cases of lymphatic filariasis (LF) [4]. Similarly, in 2013, DR Congo had the largest number of cases of onchocerciasis and human African trypanosomiasis and the third largest number of cases of schistosomiasis [4]. Both nations lead Africa in terms of numbers of cases of human hookworm infection and other NTDs [4]. A repeat analysis using information from the GBD 2017 reveals that the NTDs situation in Nigeria and DR Congo remains just as dire, but now Nigeria has overtaken both India and Indonesia in terms of the nation with the largest numbers of LF cases, while Nigeria and DR Congo may now have the world’s highest incident number of cases of malaria [5]. The consequences of the geographic overlap and coendemicity of schistosomiasis, human hookworm infection, and malaria have been reported previously and include profound and incapacitating anemia, especially among children and women of reproductive age [6–9]. In addition to its important maternal–child health consequences, anemia also profoundly affects economic productivity [10]. We therefore can anticipate that anemia from the syndemic NTDs and malaria will continue to be a dominant theme for Nigeria and DR Congo in the 21st century.

While extreme poverty is probably the dominant factor in the persistence of NTDs in Nigeria and DR Congo, other drivers have been noted, including political instability and conflict in the region [11, 12]. Although the mechanisms underlying these two factors on health require further study, undoubtedly, their ability to collapse health systems is paramount. We’re now seeing this play out in North Kivu of Eastern DR Congo, where conflict hinders an international effort to control Ebola virus infection despite the availability of a new vaccine [13, 14]. Our analyses find that conflict may rank second only to poverty as a modern driver of the NTDs [15]. Still other factors promoting the NTDs are malnutrition and food insecurity and climate change, especially for vector-borne NTDs [16].

In the coming decades, urbanization will become yet another important driver for NTDs in Nigeria and DR Congo. According to some projections, by the year 2050, Kinshasha in DR Congo will be the world’s fourth largest city, with an estimated population of 35 million people, while Lagos, with over 32 million people, will be the sixth largest [17]. Among the consequences of urbanization—working together with poverty, food insecurity, climate change, and political instability—will be new urban foci of hookworm (and other soil-transmitted helminth infections), schistosomiasis, and possibly onchocerciasis; arbovirus infections transmitted by *Aedes aegypti*; canine rabies; and selected bacterial infections such as leptospirosis, cholera, and typhoid fever, to name a few [18].

There are concerns that the emerging megacities in Nigeria and DR Congo could present special challenges for the control of NTDs. In the setting of extreme poverty, rapid urbanization and population growth can be expected to outstrip basic infrastructures related to water, sanitation, and hygiene (WASH) and food security [19] such that high levels of NTD transmission might be sustained.

As we head towards 2050, the global health community will need to respond to the continued emergence of NTDs and malaria in DR Congo and Nigeria. Of particular relevance might be the impact of current approaches to malaria control that rely on antimalarial drugs and bednets or for NTDs dependent on preventive chemotherapy, which might not be able to keep up with the intense pressures associated with large populations affected by sustained disease transmission. In the projected megacities of DR Congo and Nigeria, new biotechnologies, including genetically modified mosquitoes and antimalaria or anthelmintic vaccines for hookworm, schistosomiasis, and onchocerciasis might find important new uses [10, 20]. Vaccinating against the syndemic anemias might be particularly important and could include multivalent vaccines that simultaneously target malaria, hookworm, and schistosomiasis [21], as well as vaccines to prevent arbovirus infections, viral hemorrhagic fevers, and bacterial diarrheal infections, among other emerging viral and bacterial diseases.

We can already project that DR Congo and Nigeria, especially their emerging megacities, will become new epicenters of the NTDs. Now is the time to embark on long-term strategies for disease control, including the prioritization of the new biotechnologies that will be needed.
